# Supplementary material for: Assessing university guidance and tutoring in higher education: Validating a questionnaire on Ecuadorian students
Source: PLoS One. 2021 Jun 15;16(6):e0253400. doi: 10.1371/journal.pone.0253400 (PMC8205179; doi:10.1371/journal.pone.0253400)
Supplement: S1 Appendix — (DOCX) [file pone.0253400.s001.docx]

**Cuestionario para la Evaluación de la Orientación y la Tutoría en Educación Superior (Q-AGT)**

Valore el grado de acuerdo con las afirmaciones que se presentan sobre la orientación y tutoría en su universidad. Marca con una **X** el número de la escala que mejor refleje su apreciación:

| Totalmente en desacuerdo | En desacuerdo | Neutro | De acuerdo | Totalmente de acuerdo |
| --- | --- | --- | --- | --- |
| **0** | **1** | **2** | **3** | **4** |

| **Ítem original** | **Ítem final** | **¿Para qué cree usted que es importante la tutoría universitaria?** | | | | | |
| --- | --- | --- | --- | --- | --- | --- | --- |
| Ítem 1 | IMP1 | Proporcionarme información sobre la organización y la estructura del centro además del plan de estudios | 0 | 1 | 2 | 3 | 4 |
| Ítem 2 | IMP2 | Facilitarme la adaptación e integración a la facultad y universidad | 0 | 1 | 2 | 3 | 4 |
| Ítem 3 | IMP3 | Ayudarme en mi desarrollo académico | 0 | 1 | 2 | 3 | 4 |
| Ítem 4 | IMP4 | Guiarme en mi carrera profesional (desarrollo profesional) | 0 | 1 | 2 | 3 | 4 |
| Ítem 5 | IMP5 | Favorecer mi desarrollo personal (fomentar la autonomía, autoestima e identidad) | 0 | 1 | 2 | 3 | 4 |
| **Ítem original** | **Ítem final** | **¿Qué requiere usted de la tutoría universitaria?** | | | | | |
| Ítem 6 | DEM1 | Información | 0 | 1 | 2 | 3 | 4 |
| Ítem 7 | DEM2 | Seguimiento académico | 0 | 1 | 2 | 3 | 4 |
| Ítem 8 | DEM3 | Orientación en la carrera profesional | 0 | 1 | 2 | 3 | 4 |
| Ítem 9 | DEM4 | Orientación sobre la inserción laboral | 0 | 1 | 2 | 3 | 4 |
| Ítem 10 | DEM5 | Orientación personal | 0 | 1 | 2 | 3 | 4 |
| Ítem 11 | DEM6 | Solución de problemas y dificultades | 0 | 1 | 2 | 3 | 4 |
| Ítem 12 | DEM7 | Toma de decisiones | 0 | 1 | 2 | 3 | 4 |
| Ítem 13 | DEM8 | Ayuda en las transiciones de nivel | 0 | 1 | 2 | 3 | 4 |
| Ítem 14 | DEM9 | Atención a los alumnos con discapacidad y con necesidades especiales | 0 | 1 | 2 | 3 | 4 |
| **Ítem original** | **Ítem final** | **¿Qué características y competencias profesionales espera usted que tenga su tutor/a?** | | | | | |
| Ítem 15 | COM1 | Conocimientos generales sobre tutoría universitaria | 0 | 1 | 2 | 3 | 4 |
| Ítem 16 | COM2 | Conocimientos sobre la estructura y organización de la carrera, así como de la Universidad en general (servicios, becas, oferta de actividades...) | 0 | 1 | 2 | 3 | 4 |
| Ítem 17 | COM3 | Conocimientos sobre la inserción social y laboral de su carrera | 0 | 1 | 2 | 3 | 4 |
| Ítem 18 | COM4 | Conocimientos sobre las técnicas propias de la tutoría (entrevistas, cuestionarios…) | 0 | 1 | 2 | 3 | 4 |
| Ítem 19 | COM5 | Características personales (empático, paciente, resolutivo, cordial, mediador, constructivo. …) | 0 | 1 | 2 | 3 | 4 |
| Ítem 10 | COM6 | Buenas relaciones intra e interpersonales | 0 | 1 | 2 | 3 | 4 |
| Ítem 21 | COM7 | Saber hacer y aceptar críticas | 0 | 1 | 2 | 3 | 4 |
