# Supplementary material for: Assessing university guidance and tutoring in higher education: Validating a questionnaire on Ecuadorian students
Source: PLoS One. 2021 Jun 15;16(6):e0253400. doi: 10.1371/journal.pone.0253400 (PMC8205179; doi:10.1371/journal.pone.0253400)
Supplement: S2 Appendix — (DOCX) [file pone.0253400.s002.docx]

**Questionnaire for the Assessment of Guidance and Tutoring in Higher Education (Q-AGT)**

Assesses the degree of agreement with the statements that are presented about guidance and tutoring at your university. Mark with an X the number on the scale that best reflects your appreciation:

| Strongly disagree | In disagreement | Unsure | Agree | Strongly agree |
| --- | --- | --- | --- | --- |
| **0** | **1** | **2** | **3** | **4** |

| **Original item** | **Final**  **item** | **Why do you think university tutoring is important?** | | | | | |
| --- | --- | --- | --- | --- | --- | --- | --- |
| Item 1 | IMP1 | Provides me with information about the organization and structure of the center in addition to the curriculum | 0 | 1 | 2 | 3 | 4 |
| Item 2 | IMP2 | Assists me in adapting to and integrating with the faculty and university | 0 | 1 | 2 | 3 | 4 |
| Item 3 | IMP3 | Helps me in my academic development | 0 | 1 | 2 | 3 | 4 |
| Item 4 | IMP4 | Guides me in my professional career (professional development) | 0 | 1 | 2 | 3 | 4 |
| Item 5 | IMP5 | Promotes my personal development (promotes autonomy, self-esteem and identity) | 0 | 1 | 2 | 3 | 4 |
| **Original item** | **Final**  **item** | **What do you demand of university tutoring?** | | | | | |
| Item 6 | DEM1 | Information | 0 | 1 | 2 | 3 | 4 |
| Item 7 | DEM2 | Academic monitoring | 0 | 1 | 2 | 3 | 4 |
| Item 8 | DEM3 | Guidance in my professional career | 0 | 1 | 2 | 3 | 4 |
| Item 9 | DEM4 | Guidance with job placement | 0 | 1 | 2 | 3 | 4 |
| Item 10 | DEM5 | Personal orientation | 0 | 1 | 2 | 3 | 4 |
| Item 11 | DEM6 | Troubleshooting and difficulties | 0 | 1 | 2 | 3 | 4 |
| Item 12 | DEM7 | Decision making | 0 | 1 | 2 | 3 | 4 |
| Item 13 | DEM8 | Helps with level transitions | 0 | 1 | 2 | 3 | 4 |
| Item 14 | DEM9 | Attention to students with disabilities and special needs | 0 | 1 | 2 | 3 | 4 |
| **Original item** | **Final**  **item** | **What professional skills should a tutor have?** | | | | | |
| Item 15 | COM1 | General knowledge about college tutoring | 0 | 1 | 2 | 3 | 4 |
| Item 16 | COM2 | Knowledge about the structure and organization of the degree course, as well as the University in general (services, scholarships, activities...) | 0 | 1 | 2 | 3 | 4 |
| Item 17 | COM3 | Knowledge about the social and work possibilities of your degree | 0 | 1 | 2 | 3 | 4 |
| Item 18 | COM4 | Knowledge of tutoring techniques (interviews, questionnaires ...) | 0 | 1 | 2 | 3 | 4 |
| Item 19 | COM5 | Personal characteristics (empathetic, patient, decisive, cordial, mediator, constructive.…) | 0 | 1 | 2 | 3 | 4 |
| Item 10 | COM6 | Good intra- and interpersonal relationships | 0 | 1 | 2 | 3 | 4 |
| Item 21 | COM7 | Knows how to give and accept criticism | 0 | 1 | 2 | 3 | 4 |
