# Supplementary material for: Assessing university guidance and tutoring in higher education: Validating a questionnaire on Ecuadorian students
Source: PLoS One. 2021 Jun 15;16(6):e0253400. doi: 10.1371/journal.pone.0253400 (PMC8205179; doi:10.1371/journal.pone.0253400)
Supplement: Attachment — Submitted filename: Response to Reviewers.pdf [file pone.0253400.s003.pdf]

Response to Reviewers

Dear Reviewers,

Firstly, we would like to thank you for the suggestions made by the Academic Editor and the Reviewers. We have dealt with all the suggestions and they have been included in the manuscript. We thank the Reviewers for their indications, which have provided our research with better understanding and higher quality.

International studies have been included in the theoretical framework. In this regard, we have added two studies (7 and 8) which are considered of interest and in line with topic under consideration. These studies had not been included in the initial version of the manuscript because they have recently been published (April 2021). These publications are very recent and high impact, so we consider that the subject is original, novel and current.

Regarding the Reviewer's question 1 related to the Likert scale, the authors clarify that the scale has 5 response options with a graduation from “0” to “4”: where “0” is “completely disagree”, “1” is "disagree", "2" is "neutral", "3" is "agree", and "4" is "completely agree". A different graduation could have been indicated, for example from “1” to “5”, but it was decided to start with “0” considering the previous studies published by the authors (e.g., Dios et al., 2018. Validation of the Scale of Organizational and Didactic Competencies for Educators). We think that both options are acceptable and they do not determine the results obtained or the validation of the scale.

Many references are in Spanish since it is the language of the country where the scale has been validated. Although the review includes international studies in Spanish and English, the language of publication in Latin America, and specifically in Ecuador, is Spanish. Most literature about counseling and tutoring in this country is published in Spanish. However, as indicated by the Academic Editor and Reviewer 1, we have included international studies published in English in the new version of the manuscript.

In response to suggestions from Reviewer 1, the sociodemographic data have been analyzed and cross-referenced with the 21 items. It has been reflected in table 3 in the results section "Application of Q-AGT: assessment of university tutoring based on sex, university and university degree course". The reference number 12 has been included; “Authors, 2017” is substituted by “Amor MI, Dios I. Analysis of the psychometric

properties of a scale on the needs of students in Tutoring. Revista de Pedagogía. 2017; 38: 35-56 ". The reference 12 in the original manuscript is now the reference 14.

The errata indicated by Reviewer 2 has been corrected. The word "express" has been deleted.

Finally, we include a manuscript called “Revised Manuscript with Track Changes”, which contains the changes made to the original version and another version called “Manuscript” with the incorporated changes.

We have made sure to check that the manuscript complies with the PLOS ONE style requirements. Besides, the figures have been uploaded and downloaded to the Preflight Analysis and Conversion Engine (PACE). We provide figure in this format. We have also included the supporting information section in the final manuscript.

As it has been indicated by the Academic Editor, we have included the title page in the main document. We have checked that the reference list is complete and correct. The changes incorporated in the reference list have been three. We have included two references to international scientific studies, recommended by the Editor. Reference 14 (reference 12 in the original manuscript) which was incomplete (indicated by Reviewer 2) has been included.

We appreciate the contributions indicated.

Yours sincerely
